# High Frequency of Pancreatic Exocrine Insufficiency Among Patients with Abdominal Pain, Bloating, and Malodorous Flatus: Hidden Pancreatic Insufficiency

**DOI:** 10.5152/tjg.2026.25746

**Published:** 2026-05-18

**Authors:** Gizem Sultan Kürtül, Orhan Sezgin, Damla Hazal Sucu

**Affiliations:** 1 Department of Internal Medicine, Mersin University Faculty of Medicine, Mersin, Türkiye; 2 Division of Gastroenterology, Department of Internal Medicine, Mersin University Faculty of Medicine, Mersin, Türkiye; 3 Department of Biostatistics, Mersin University Faculty of Medicine, Mersin, Türkiye

**Keywords:** Abdominal pain, bloating, fecal elastase-1, malodorous flatus, pancreatic exocrine insufficiency

## Abstract

**Background/Aims:**

Pancreatic exocrine insufficiency (PEI) is a condition characterized by impaired digestion and absorption resulting from insufficient exocrine secretion of the pancreas. The current study aimed to determine the prevalence, clinical characteristics, and associated risk factors of PEI in patients presenting to the gastroenterology outpatient clinic with common gastrointestinal complaints such as abdominal pain, dyspepsia, and defecation disorders.

**Materials and Methods:**

Three hundred ten symptomatic patients were retrospectively evaluated using demographic data, biochemical markers, the PEI Questionnaire, and fecal elastase-1 (FE-1) levels. PEI was defined as an FE-1 level <200 µg/mL, with levels <100 µg/mL indicating severe deficiency.

**Results:**

PEI was identified in 45.8% (n = 142) of the cohort, with 43% classified as severe. PEI was significantly more common in males (*P *= .024). Key comorbidities associated with PEI included diabetes mellitus, hypertension, hyperlipidemia, and prior gastric or pancreatic surgery. Abdominal pain, bloating, and particularly malodorous flatus (odds ratio = 3.705) were strong predictors of PEI. Severe PEI was associated with significantly lower hemoglobin and vitamin D levels. Sixty-five percent of patients with PEI had prior symptoms but had not been diagnosed.

**Conclusion:**

PEI is highly prevalent among patients with non-specific gastrointestinal (GI) symptoms and is frequently underdiagnosed. Clinicians should maintain a high index of suspicion, particularly in patients with metabolic comorbidities or prior GI surgeries.

**Main Points**

- Nearly half of the patients presenting with abdominal pain, bloating, and malodorous flatus had pancreatic exocrine insufficiency (PEI).
- Malodorous flatus is the strongest symptomatic predictor of PEI and may represent a red flag.
- Severe PEI is associated with decreased hemoglobin and vitamin D levels, highlighting the need for early intervention to prevent long-term morbidity.
- Fecal elastase-1 testing and the Pancreatic Exocrine Insufficiency Questionnaire should be integrated into the diagnostic workup for patients with persistent “functional” gastrointestinal symptoms, particularly those with metabolic comorbidities or prior gastric or pancreatic surgery.

## Introduction

Pancreatic exocrine insufficiency (PEI) involves a deficiency in pancreatic enzyme secretion or activity, leading to nutrient maldigestion and subsequent malabsorption.^1^^,^^2^ It can impair quality of life and result in significant morbidity.^3^^,^^4^ While its association with chronic pancreatitis and cystic fibrosis is well established, its prevalence among patients presenting with general dyspeptic or irritable bowel syndrome (IBS)-like symptoms remains poorly characterized.^5^

The diagnosis of PEI is often delayed because its symptoms, including abdominal pain, bloating, and flatulence, significantly overlap with those of functional gastrointestinal disorders. Fecal elastase-1 (FE-1) remains a non-invasive gold standard for screening because of its stability during intestinal transit.^5^^-^^8^ The current study aimed to determine the prevalence of PEI and identify clinical predictors that may facilitate earlier diagnosis in an outpatient setting.

## Materials and Methods

This retrospective cohort study was conducted at the Department of Gastroenterology, Mersin University Faculty of Medicine, and included patients evaluated by the same investigator (OS) between January 2018 and February 2024. Ethical committee approval was obtained from the Ethics Committee of Mersin University (Approval no: 78017789-050.01.04-2665318 Date: March 6, 2024).

Consecutive patients aged 18 years or older with complaints such as abdominal pain, bloating, gas, malodorous flatus, and defecation disturbances were included in the study. Detailed clinical evaluation, physical examination, and laboratory workup, including FE-1 testing (Immundiagnostik AG, Bensheim, Germany), were performed. Abdominal ultrasonography, and, when indicated, upper and lower gastrointestinal (GI) endoscopy and advanced imaging were also performed to exclude other organic pathologies. Results of the Pancreatic Exocrine Insufficiency Questionnaire (PEI-Q), a patient-completed diagnostic survey,^9^ were recorded. PEI was defined as an FE-1 level <200 µg/mL.^3^ Based on FE-1 levels, severity was classified as mild to moderate (100-200 µg/mL) and severe (<100 µg/mL). Associations between PEI and gastrointestinal symptoms and comorbidities, as well as differences between mild to moderate and severe PEI, were analyzed. To minimize the false-positive results in FE-1 testing, measurements were repeated every 3-6 months during the study. Patients with normal FE-1 levels on repeat testing were classified as PEI negative. Given the retrospective design of the study, informed consent from the patients could not be obtained.

### Statistical Analysis

Data were analyzed using TIBCO Software STATISTICA, Version 13.5.0.17 (TIBCO; CA, USA). Descriptive statistics are presented as mean, SD, median, median of first quartile to third quartile, count, and percentage. Associations between categorical variables were evaluated using the chi-square test. For contingency tables larger than 2 × 2 with statistically significant results, the *Z*-test was used to identify the groups contributing to the difference. For comparisons between 2 groups, the Student’s *t*-test was used when the normality assumption was met; otherwise, the Mann–Whitney *U*-test was applied. For comparisons among more than 2 independent groups, the Kruskal–Wallis test was used. Associations between continuous variables were assessed using Pearson correlation. When the dependent variable was continuous, linear regression was used to assess its association with independent variables. When the dependent variable was binary, logistic regression analysis was conducted to model the influencing factors. The diagnostic discriminatory power was evaluated through receiver operating characteristic (ROC) curve analysis. To explore the relationships and structural connections among variables, link analysis was performed, and the results were visualized using a web graph. A *P* value <.05 was considered statistically significant.

## Results

### Frequency and Demographics

**Figure 1.**
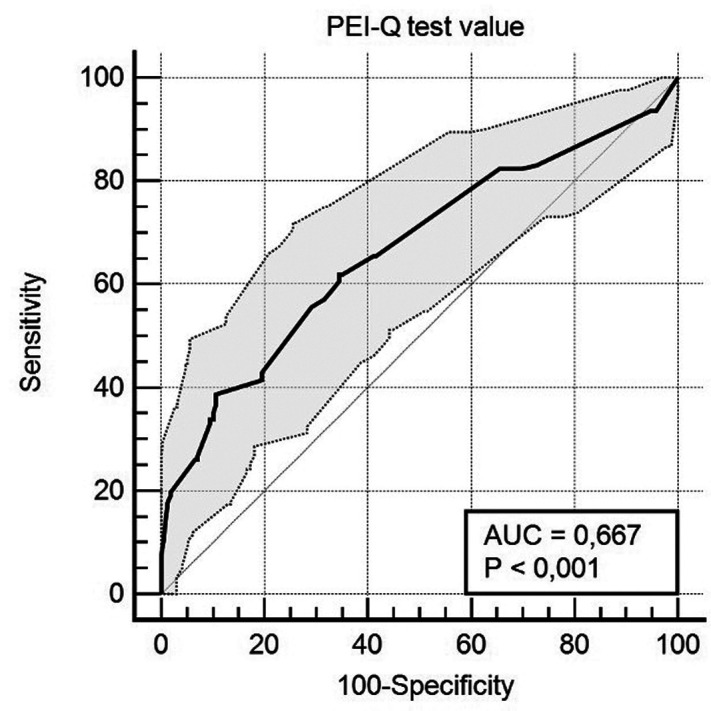
ROC analysis of the predictive power of the PEI-Q test for those with and without PEI. AUC, area under the curve; PEI-Q, Pancreatic Exocrine Insufficiency Questionnaire; ROC, receiver operating characteristic curve.

Of 310 patients, 142 (45.8%) tested positive for PEI. Detailed characteristics of these patients are presented in Table 1. PEI was significantly more prevalent in male patients than in female patients (59.9% vs. 40.1%, *P *= .024). Among PEI-positive patients, PEI-Q test scores, hemoglobin A1c levels, and systolic blood pressure values were significantly higher than in PEI-negative patients. The discriminating performance of the PEI-Q test for detecting PEI was evaluated using ROC analysis. The values were as follows: sensitivity (CI) = 38.73 (30.7-47.3), specificity (CI) = 89.29 (83.6-93.5), positive predictive value (PPV) (CI) = 75.3 (65.3-83.2), negative predictive value (NPV) (CI) = 63.3 (59.7-66.2), *P *< .001, area under the curve (AUC) (CI) = 0.667 (0.605-0.728) (Figure 1). The analysis revealed the statistically significant discriminating performance of PEI-Q in differentiating patients with and without PEI. The median PEI-Q score was 0.85 (0.57-1.18) and 0.57 (0.28-0.85) in PEI-positive and -negative patients without diabetes, respectively (*P* < .001), whereas 0.57 (0.28-1.07) and 0.57 (0.37-0.75) in PEI-positive and -negative patients with diabetes, respectively (*P *= .193).

### Comorbidities and Surgical History

Patients with PEI had a significantly higher frequency of metabolic conditions as follows:

- Diabetes mellitus: 27.5% in the PEI group vs. 14.9% in the PEI-negative group (*P *= .006).
- Hypertension and hyperlipidemia: Both were significantly more common in the PEI-positive cohort (*P *= .010 and *P* = .003, respectively).
- Surgery: A strong correlation was observed with prior gastric (*P *= .016) and pancreatic surgeries (*P *= .032). Of the 8 patients who had undergone gastric surgery, 7 had PEI. Of these 7 patients, 3 had a history of the Whipple procedure, 2 had laparoscopic Nissen fundoplication, 1 had distal subtotal gastrectomy, and 1 had undergone gastric bypass surgery. Six of the 7 patients who had undergone pancreatic surgery had PEI.

No statistically significant differences were observed between the groups in anthropometric measurements and lifestyle factors (alcohol and smoking) (Table 2).

### Severity of PEI

**Figure 2.**
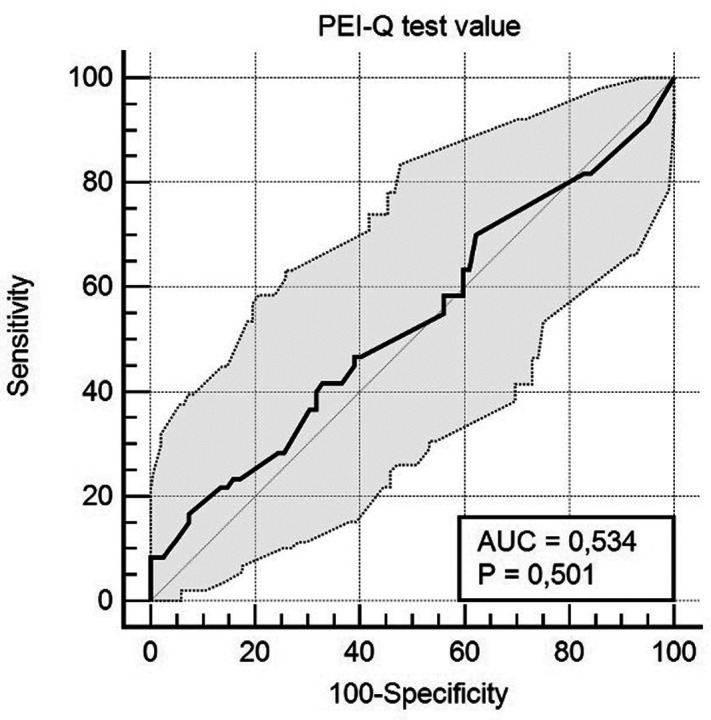
ROC analysis of the predictive power of the PEI-Q test in demonstrating the severity of PEI. AUC, area under the curve; PEI-Q, Pancreatic Exocrine Insufficiency Questionnaire; ROC, receiver operating characteristic curve.

Forty-three percent of the patients with PEI suffered from severe insufficiency (FE-1 <100 µg/mL). Detailed characteristics of patients with severe and mild-to-moderate PEI are summarized in Table 2. The only difference between the 2 groups was low hemoglobin and vitamin D levels in the severe group. PEI-Q scores were similar in the mild-to-moderate and severe groups. The performance of the PEI-Q test in differentiating PEI severity was evaluated. The results of ROC analysis were as follows: AUC (CI) = 0.534 (0.436-0.632), sensitivity (CI) = 16.67 (8.3-28.5), specificity (CI) = 92.68 (84.8-97.3), *P *= .501 (Figure 2). The discriminative performance of PEI-Q was found to be non-significant.

The degree of PEI was similar in patients with and without diabetes (mild-to-moderate PEI: 53.8% vs. 59.2%, severe PEI: 46.2% vs. 40.8%, respectively *P *= .563).

Factors influencing the severity of PEI, such as BMI, alcohol, smoking, diabetes, and stool consistency, were evaluated using multivariate logistic regression. Alcohol consumption alone was identified as a strong and independent risk factor for severe PEI, with an approximately 6.5-fold increase in risk.

### Symptom Overlap

The association between the presence of PEI and gastrointestinal symptoms was evaluated in the study sample. Abdominal pain (71.8% vs. 52.4%, *P *< .001), abdominal bloating (62.7% vs. 50.0%, *P *= .025), and malodorous flatus (8.5% vs. 2.4%, *P *= .016) were significantly more prevalent in patients with PEI than in those without PEI. There was no significant association with symptoms including flatulence (43.0% vs. 35.1%, *P *= .158), diarrhea (35.2% vs. 31.0%, *P *= .427), constipation (18.3% vs. 22.6%, *P *= .350), weight loss (21.8% vs. 14.3%, *P *= .083), nausea (25.4% vs. 20.2%, *P *= .283), and loss of appetite (13.4% vs. 11.3%, *P *= .580).

Logistic regression analysis demonstrated that malodorous flatus was associated with a 3.7-fold increased risk (odds ratio (OR) = 3.705, *P *= .031), abdominal pain with a 2.3-fold increased risk (OR=2.307, *P *= .001), and abdominal bloating with a 1.6-fold elevation risk (OR = 1.667, *P *= .032).

**Figure 3.**
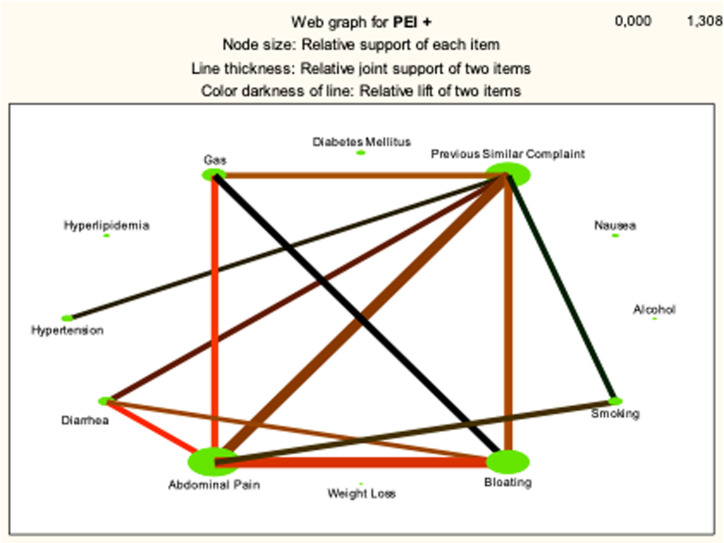
Symptoms associated with complaints of PEI patients and the relationships between these symptoms—Link analysis (web graph). PEI, pancreatic exocrine insufficiency.

The results of the link analysis, presented in the web graph (Figure 3), revealed the associations between symptoms and comorbidities in the PEI patients cohort. In this analysis, “previous similar complaints,” “abdominal pain,” “bloating,” and “gas” emerged as the most prevalent clinical findings. The thickness and color intensity of the connections between these symptoms indicate a statistically significant tendency for co-occurrence. When evaluated in terms of comorbidities, metabolic and cardiovascular pathologies such as Diabetes mellitus (DM), Hyperlipidemia (HL), and Systemic arterial hypertension (HT) were found to be associated with PEI. However, the relatively smaller size of the nodules suggests that these comorbidities are less prevalent than gastrointestinal symptoms. In terms of etiological risk factors, “alcohol” and “smoking” are represented as peripheral nodes in the graph, and the thickness and color intensity of their connections with other clinical parameters suggest that these factors may play a role as risk factors in patients with PEI. This link analysis demonstrated that gastrointestinal symptoms constitute the dominant clinical picture in patients with PEI, exhibit strong interrelationships, and may be accompanied by metabolic comorbidities.

A statistically significant proportion (65%) had a history of similar chronic complaints but had not previously received a diagnosis. In contrast, this proportion was 29.2% in the PEI-negative group (*P *< .001).

**Figure 4.**
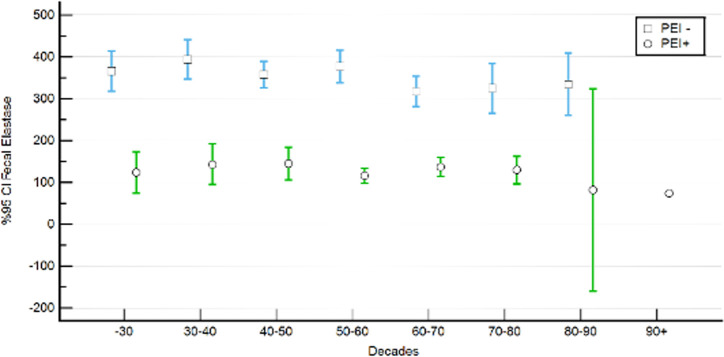
Mean focal elastase (μ/mL) levels of all patients stratified by age decades.

To evaluate the association between PEI and age, patients were grouped by decade. FE-1 levels were compared across age groups in both cohorts. No statistically significant differences in FE1 levels were observed across decades in either the PEI-negative or PEI-positive groups (*P * = .139 and *P * = .767, respectively) (Figure 4).

PEI-Q scores of patients with PEI were analyzed according to age decades. No statistically significant difference was found in mean PEI-Q scores across the decades (third decade: 0.77 ± 0.44, fourth decade: 0.85 ± 0.46, fifth decade: 0.78 ± 0.38, sixth decade: 0.68 ± 0.41, seventh decade: 0.61 ± 0.34, eighth decade: 0.64 ± 0.52, ninth decade: 0.69 ± 0.13; *P * = .064).

## Discussion

The findings of the current study suggest that PEI is a “hidden” diagnosis in approximately 45% of patients presenting with common GI discomfort. This is notably higher than the rates reported in studies specifically targeting populations with dyspepsia or IBS. Forty-three percent of these patients had severe PEI, with low vitamin D and hemoglobin levels. The association with metabolic conditions, particularly diabetes and hypertension, highlights the importance of recognizing PEI.

PEI is a clinical condition characterized by maldigestion and malabsorption resulting from impaired pancreatic enzyme activity.^10^ This may be because of insufficient production of pancreatic enzymes, inadequate delivery of enzymes to the intestine, insufficient enzyme activation, or premature enzymatic degradation.^5^The primary clinical consequence of PEI is malnutrition resulting from impaired absorption of nutrients. Nutritional deficiencies typically include protein, fat-soluble vitamin, and micronutrient deficiencies, small intestinal bacterial overgrowth, and significant dysbiosis of the gut microbiota.^11^^,^^12^Complications of malnutrition include cardiovascular events, weight loss, osteoporosis, sarcopenia, fall-related fractures, and increased susceptibility to infections.^13^ Regardless of the underlying etiology of PEI, gastrointestinal symptoms and nutritional deficiencies can impair quality of life and increase the risk of long-term malnutrition-related complications, thereby contributing to increased morbidity and mortality.^14^

Maldigestion and inadequate nutrient absorption resulting from PEI commonly lead to gastrointestinal symptoms such as abdominal pain, bloating, diarrhea, altered bowel habits, and excessive gas. These symptoms are among the most common reasons for presentation to general practice and gastroenterology outpatient clinics.

For example, in the community-based “Cappadocia Cohort Study” conducted in Türkiye by Sezgin et al,^15^ at least 1 GI symptom was reported in 70% of the population. The most commonly reported upper GI symptoms included abdominal bloating (31.0%), heartburn (29.1%), belching (23.8%), epigastric pain (21.4%), and halitosis (19.7%). The most commonly reported lower GI symptoms were defecation disturbances (33.5%), borborygmi (24.2%), abdominal distension (22.2%), and abdominal pain (16.2%). These findings are very similar to those observed in patients with PEI. Patients presenting with these symptoms are often diagnosed with functional gastrointestinal disorders such as IBS, functional bowel disease, or dyspepsia. In routine clinical practice, PEI is rarely considered as a primary diagnosis, potentially delaying accurate diagnosis and appropriate management for a significant period. In the current study, 65% of patients with PEI had previously experienced these symptoms; however, the possibility of a PEI diagnosis had not been considered or evaluated. Delayed diagnosis of PEI is a significant disadvantage for patients, as the adverse effects of the condition progressively worsen over time.

A logistic regression analysis showed that certain symptoms were significant indicators of PEI positivity: malodorous flatus was associated with a 3.7-fold increased risk (OR = 3.705, *P *= .031), abdominal pain with a 2.3-fold increased risk (OR = 2.307, *P *= .001), and abdominal bloating with a 1.6-fold increased risk (OR = 1.667, *P *= .032). These findings suggested that the aforementioned gastrointestinal symptoms, particularly malodorous flatus, may serve as clinically meaningful predictors in the diagnosis of PEI. In PEI, undigested foods, particularly fats, undergo fermentation in the intestines, leading to the production of gas and bloating.

Because of its limited specificity, PEI cannot be diagnosed solely based on the symptoms that are specifically observed; pancreatic function tests and nutritional deficiencies are not unique to PEI. Although FE-1 is a monoclonal antibody specific to human pancreatic elastase that remains stable during intestinal transit and reflects exocrine functional capacity, there is a possibility of false positives.^8^ Therefore, diagnosis should be based on a combined evaluation of symptoms, nutritional status, and pancreatic function. In 2018, Johnson et al^9^ developed the PEI-Q, a symptom-based, patient-completed questionnaire designed to assist in the diagnosis of PEI. A PEI-Q score cutoff of >0.80 was found to significantly differentiate patients with PEI from healthy controls. Subsequently, the psychometric validation and reliability of this tool were established.^16^ Oğuz et al^17^ reported that the Turkish version of the PEI-Q is a reliable, easy-to-use, and valid screening tool. Therefore, the current study evaluated the combined effects of symptoms, FE-1 testing, and the PEI-Q.

The PEI-Q results showed that it was effective in distinguishing patients with PEI from those without, but ineffective in differentiating between mild-to-moderate PEI and severe PEI. The similarity in PEI-Q scores indicated no association between PEI severity and symptoms. While the PEI-Q test can differentiate the presence of PEI in patients without diabetes, it cannot distinguish it in patients with diabetes.

There is insufficient information regarding the frequency of PEI in the general population and in patients with GI symptoms. The prevalence of PEI has been extensively investigated in studies conducted in at-risk populations, such as individuals with DM, chronic pancreatitis, or those who have undergone pancreatic surgery. Few studies have investigated the prevalence of PEI in patients with persistent dyspepsia or IBS. Tahtaci et al^18^ reported a significantly higher percentage of lower FE1 levels in a small group of patients with non-ulcer dyspepsia. Other studies have also detected varying rates of PEI in patients with dyspepsia, albeit using different methods.^19^^-^^22^ In a few studies involving patients with IBS, the prevalence of PEI was reported to be 5% and 6.1%.^23^^,^^24^Talley et al^25^ reported a prevalence of PEI of 4.6% in patients with unexplained abdominal pain or diarrhea. In the current study, 45% of patients presenting to the gastroenterology clinic with gastrointestinal discomfort, particularly abdominal pain, bloating, and malodorous flatus, were diagnosed with PEI.

In the current study, 43% of the patients with PEI had severe disease, characterized by significantly low hemoglobin and vitamin D levels. Mann et al^26^ previously reported a significant correlation between low FE-1 levels and 25(OH)D(3), 1,25(OH)_2_D(3), calcium, and parathyroid hormone levels.The association between severe PEI and low vitamin D and hemoglobin levels is a key finding. This suggests that by the time PEI is diagnosed in these patients, it has already progressed to a stage of biochemical malabsorption, potentially increasing the risk of osteoporosis and anemia.

The authors detected PEI in 61% of patients with diabetes and 42% of patients without diabetes (*P =* .006). The frequency of DM was significantly higher in patients with PEI than in those without PEI. These findings highlight the importance of the relationship between PEI and DM. PEI has perhaps been most extensively studied in diabetic populations. In a meta-analysis published by Zsóri et al,^27^ PEI was found in 52.4% of diabetic patients. The frequency of HT and HL was also significantly higher in PEI-positive patients. There is a lack of specific or comprehensive data on the prevalence of HT and HL in patients with PEI, as well as the exact mechanisms underlying these relationships. De la Iglesia et al^3^ reported a higher incidence of cardiovascular events in patients with chronic pancreatitis and PEI, with multivariable analyses identifying PEI as a risk factor associated with cardiovascular events such as smoking and HT.^4^ The metabolic link, particularly with diabetes and hypertension, underscores that PEI is not just a localized digestive issue but often part of a broader metabolic or cardiovascular risk profile. This supports prior research suggesting that PEI may be a risk factor for cardiovascular events. Because of these reasons, PEI warrants further investigation in relation to cardiovascular health and long-term outcomes.

PEI is a well-known complication of pancreatic and gastric surgery.^1^^,^^10^^,^^28^ In the current study, patients with PEI had a higher prevalence of pancreatic and gastric surgical histories compared to those without PEI.

Although previous studies have shown an increase in the prevalence of PEI with age, the current study did not find a significant relationship between age, aging, and PEI (Figure 4).^29^ The prevalence of PEI was higher in male patients than that in female patients (Table 1).

The current study was conducted on a homogeneous patient group under the supervision of a single researcher using a defined standard protocol. However, its retrospective nature constitutes a limitation.

In conclusion, PEI is quite common in patients presenting with gastrointestinal symptoms. These patients were more likely to experience malodorous flatus, abdominal pain, and bloating. A significant portion of patients had severe PEI and similar prior symptoms. The lower levels of vitamin D and hemoglobin in patients with severe PEI further highlight the importance of early diagnosis and treatment. For diagnosing PEI, a combined evaluation of symptoms, pancreatic function tests, and nutritional status is essential.

## Figures and Tables

**Table 1.** Comparison of Patients with and without PEI in the Study Cohort

**Variable**	**PEI Patients**	**PEI Negative**	***P***
Age, mean ± SD	54.0 ± 14.9	52.5 ± 16.0	.383**
Gender, n (%) Female Male	57 (40.1) 85 (59.9)	89 (53.0) 79 (47.0)	.024
PEI-Q test score, median	0.85	0.57	<.001*
Height (cm), median	170	168	.158*
Weight (kg), mean ± SD	78.9 ± 18.0	75.0 ± 17.0	.087**
BMI, median	27.1	26.4	.131*
BMI, n (%) 18.5-24.9 25.0-29.9 30+	45 (39.1) 37 (32.2) 33 (28.7)	37 (34.3) 33 (30.6) 38 (35.2)	.565
Smoking, n (%)	47 (33.1)	49 (29.2)	.456
Alcohol, n (%)	32 (22.5)	26 (15.5)	.112
Systolic BP (mmHg), median	125	120	.013*
Diastolic BP (mmHg), median	80	80	.102*
Fasting glucose (mg/dL), median	102	98	.103*
HbA1c (%), median	6.00	5.50	<.001*
ALT (U/L), median	21.00	20.00	.692*
Total cholesterol (mg/dL), mean ± SD	201.7 ± 50.9	200.4 ± 53.9	.847**
Triglycerides (mg/dL), median	132.5	122	.392*
HDL cholesterol (mg/dL), median	50	49	.251*
LDL cholesterol (mg/dL), median	115.2	117.6	.460*
Hemoglobin (g/dL), median	14	14	.925*
Leukocyte count (µL), median	7.4	7.1	.115*
Platelet count (µL), mean ± SD	268.71 ± 92	262.39 ± 81	.550**
C-reactive protein (mg/dL), median	2.1	2.5	.913*
Vitamin D (ng/mL), median	20	23	.395*
Vitamin B12 (pg/mL), median	232	256	.429*
Ferritin (ng/mL), median	49.9	41.0	.196*
Fecal elastase (µg/mL), mean ± SD	129.47 ± 72	355.38 ± 101	<.001**
Diabetes mellitus, n (%)	39 (27.5)	25 (14.9)	.006
Hypertension, n (%)	43 (30.3)	30 (17.9)	.010
Hyperlipidemia, n (%)	35 (24.6)	20 (11.9)	.003
Chronic pancreatitis, n (%)	5 (3.5)	5 (3.0)	.787
Malignancy, n (%)	0 (0.0)	1 (0.6)	.357
Ulcerative colitis, n (%)	4 (2.9)	3 (1.8)	.530
Crohn’s disease, n (%)	4 (2.8)	4 (2.4)	.809
Celiac disease, n (%)	4 (2.8)	5 (3.0)	.934
History of pancreatic surgery, n (%)	6 (4.2)	1 (0.6)	.032
History of cholecystectomy, n (%)	14 (9.9)	29 (17.3)	.060
History of gastric surgery, n (%)	7 (4.9)	1 (0.6)	.016
Metabolic syndrome, n (%)	48 (36.6)	44 (30.8)	.304

Test: *P*, chi-square; P^*^, Mann–Whitney *U* and P ^**^, Student’s *t*, Q1, 25% percentile; Q3, 75% percentile.

ALT, alanine transaminase; BMI, body mass index; BP, blood pressure; HDL, high-density lipoprotein; LDL, low-density lipoprotein; PEI, pancreatic exocrine insufficiency.

**Table 2.** Comparison of Patients With Severe PEI and Those With Mild-to-Moderate PEI

**Variable**	**Severe PEI**	**Mild and Moderate PEI**	*P*
PEI Q Test Score, median	0.85	0.85	.492^*^
Age, mean ± SD	54.5 ± 16.9	53.7 ± 13.4	.832^**^
Gender, n (%) Female Male	29 (48.3) 31 (51.7)	28 (34.1) 54 (65.9)	.088
Height (cm), median	170	170	.281^*^
Weight (kg), mean ± SD	76.2 ± 19.0	80.9 ± 17.1	.166^**^
BMI, median	25.5	28.3	.257^*^
Systolic BP (mmHg), median	120	130	.321^*^
Diastolic BP (mmHg), median	80	80	.085^*^
Fasting glucose (mg/dL), median	109	101	.169^*^
HbA1c (%), median	6.0	5.9	.764^*^
Insulin, median	6.85	9.50	.123^*^
HOMA-IR, median	2.35	2.72	.317^*^
AST (U/L), median	19	20	.295^*^
ALT (U/L), median of Q1-Q3	18.5	23	.066^*^
Total cholesterol (mg/dL), mean ± SD	196.50 ± 53	206.00 ± 48	.310^**^
Triglycerides (mg/dL), median	112	136	.117^*^
HDL cholesterol (mg/dL), median	50	51	.820^*^
LDL cholesterol (mg/dL), median	109.4	120.9	.445^*^
Hemoglobin (g/dl), median	13.1	14.7	.003^*^
Leukocyte count (µL), median	7.1	7.4	.304^*^
Platelet count (µL), mean ± SD	275.86 ± 102.96	263.19 ± 84.53	.437^**^
C-reactive protein (mg/dL), median	2.0	2.4	.592^*^
Vitamin D (ng/mL), median	18.4	23.5	.026^*^
Vitamin B12 (pg/mL), median	222	244	.313^*^
Ferritin (ng/mL), median	50.5	46.0	.803^*^
Fecal elastase (µg/mL), mean ± SD	89.35 ± 7	158.83 ± 5	<.001^**^
Diabetes mellitus, n (%)	18 (30.0)	21 (25.6)	.563
Hypertension, n (%)	17 (28.3)	26 (31.7)	.666
Hyperlipidemia, n (%)	14 (23.3)	21 (25.6)	.756

Test: *P*, chi-square; P^*^, Mann–Whitney *U* and P^**,^ Student’s *t*; Q1, 25% percentile; Q3, 75% percentile.

ALT, alanine transaminase; AST, aspartate transaminase; BMI, body mass index; BP, blood pressure; HDL, high-density lipoprotein; HOMA-IR, homeostatic model assessment of insulin resistance; LDL, low-density lipoprotein; PEI, pancreatic exocrine insufficiency.

## Data Availability

The data that support the findings of this study are available on request from the corresponding author.
